# Coronavirus disease 2019 associated with aggressive neurological and mental abnormalities confirmed based on cerebrospinal fluid antibodies: A case report

**DOI:** 10.1097/MD.0000000000021428

**Published:** 2020-09-04

**Authors:** Maomao Wang, Ting Li, Fan Qiao, Laixing Wang, Chunlin Li, Yanping Gong

**Affiliations:** a Department of Neurosurgery, Chang Hai Hospital, The Second Military Medical University, Shanghai; b Huo Shen Shan Hospital, Wuhan; c Department of Endocrinology, The Second Medical Center, The People's Liberation Army General Hospital, Beijing, China.

**Keywords:** antibodies, cerebrospinal fluid, coronavirus disease 2019, mental abnormalities, neurological injuries

## Abstract

**Introduction:**

Coronavirus disease (COVID-19) is spreading worldwide. The reported possible neurological symptoms are varied and range from subtle neurologic deficits to unconsciousness. Knowledge regarding the detection, diagnosis, treatment, and follow-up of COVID-19-associated neurological damage is still limited. We report a case of serious neurological damage and mental abnormalities in a patient who was finally confirmed to have COVID-19 based on IgM and IgG antibodies in the cerebrospinal fluid (CSF).

**Patient concerns:**

A 68-year-old man had slight flu-like symptoms and transient loss of consciousness in early February. Exaggerated unconsciousness and deteriorating mental abnormalities occurred over the next month without severe respiratory symptoms. Craniocerebral computed tomography showed normal results, but antibodies against severe acute respiratory syndrome coronavirus 2 were 100 times higher in the CSF than in the serum; tests for viral ribonucleic acid showed negative results with both a nasopharyngeal swab and CSF sample.

**Diagnosis:**

COVID-19 pneumonia was diagnosed based on symptoms and positive results for IgM and IgG in the CSF.

**Interventions:**

Antiviral, fluid, and nutritional support were administered for 30 days before admission without obvious improvement. A further 18 days of routine antiviral therapy, immunoglobulin therapy (10 g per day for 5 days), and antipsychotic drug treatment were administered.

**Outcomes:**

The patient's neurological and mental abnormalities were greatly ameliorated. He was discharged with mild irritability, slight shaking of the hands, and walking fatigue. These symptoms have persisted up to our last follow-up (May 4, 2020).

**Conclusion:**

We believe this is the first case involving neural system injury in a patient who confirmed COVID-19 based on CSF antibody test results. Negative ribonucleic acid test results, strong positivity for antibodies, and high protein levels in the CSF suggest the possibility of autoimmune encephalitis secondary to COVID-19. This case highlights additional novel symptoms of COVID-19, and these data are important for the assessment and follow-up of COVID-19 patients.

## 1 Introduction

The novel coronavirus disease, coronavirus disease 2019 (COVID-19), has become a pandemic and is currently spreading around the world and causing concern in the medical community.^[1,2]^ Severe acute respiratory syndrome coronavirus 2 (SARS-CoV-2), the virus causing COVID-19, is different from the zoonotic Middle East respiratory syndrome (MERS) -CoV and SARS-CoV and is the seventh coronavirus to infect humans.^[1]^ Phylogenetic analysis of these coronaviruses based on full-length genome sequences shows that SARS-CoV-2 has about 45–90% similarity with SARS-CoV and 20% to 60% similarity with MERS-CoV.^[3]^ Researchers are racing to learn more about the epidemiology and clinical characteristics of this new viral infection. We report a case of serious neurological damage and mental abnormalities in a patient who was finally confirmed to have COVID-19 infection based on IgM and IgG antibodies in the cerebrospinal fluid (CSF), despite testing negative for viral ribonucleic acid (RNA) using both a nasopharyngeal swab and CSF sample; tests for plasma antibodies also showed negative results.

## 2 Case presentation

A 68-year-old man temporarily lost consciousness on February 2, 2020 with slight cough, vomiting, fatigue, and low fever (37.5°C). There was no apparent sputum production, sore throat, chest tightness, difficulty breathing, headache, or limb convulsion. Chest computed tomography (CT) showed ground-glass shadows in both lungs. He was recommended to isolate at home due to a suspicion of “new coronavirus pneumonia.” Aggravated fatigue accompanied by headache, dizziness, nausea, and coma occurred on February 8. Emergency craniocerebral CT revealed lacunar lesions in the left basal ganglia region and chest CT showed dispersive and patchy shadows bilaterally in the lungs with partial consolidation; the lesion had enlarged. The patient was clinically diagnosed with “new coronavirus pneumonia” and was hospitalized. He awoke 48 h later with headache and vomiting after antiviral treatment and fluid and nutritional support. During this period, the patient underwent 2 viral RNA tests by pharyngeal swab, both of which were negative. On February 23, the patient once again slipped into a comatose state without any apparent cause. He woke up 4 days later unable to walk and with uroclepsia, coprolalia, and persecution delusion. He was transferred to our hospital on March 3. The patient had a history of diabetes and hypertension under good control. His wife was also diagnosed with "new coronavirus pneumonia” a few days after his diagnosis. Her symptoms were mild without loss of consciousness or mental abnormalities. There was no reported history of mental disorders in the family.

**Figure 1:**
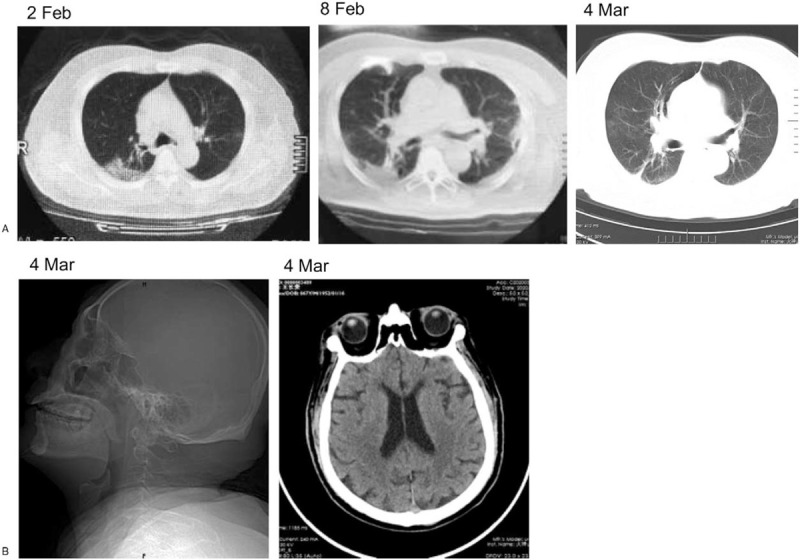
Craniocerebral and chest computed tomography (CT) of this patient. A. Chest CT changes in this patient: lesions were distinctly increased and enlarged on February 8 compared with those on February 2, and shadows and lesions were obviously decreased and ameliorated. B. Craniocerebral CT: lacunar lesions in the left basal ganglia region. CT = computed tomography.

His blood oxygen saturation maintained above 95% without supplemental oxygen even in his poor condition after admission. Slight neck stiffness, trembling of the hands, and grade 4 muscle strength were detected. Laboratory investigations showed normal liver function, renal function, electrolyte level, and coagulation function. Leucocyte and lymphocyte count and percentage were all within a reasonable range. SARS-CoV-2 IgM antibodies in the plasma were slightly high and IgG antibodies in the plasma were within the normal range (10.4 and 0.28 respectively, with normal range <10, chemiluminescence immunoassay; iFlash-SARS-CoV-IgM: C86095 M, iFlash-SARS-CoV-IgG: C86095G, YHLO Biotech, Shenzhen, China). A repeated craniocerebral CT showed results similar to those obtained on February 8; another chest CT showed ameliorated lung opacities (Fig. 1). On March 5, the cerebrospinal fluid (CSF) was strongly positive for SARS-CoV-2 IgM antibodies and IgG antibodies (107.52 and 70.07, respectively). However, his swab and CSF RNA tests were consistently negative. The protein level in the CSF was significantly high (803.6 mg/L), but no obvious abnormality was found in routine examination. Craniocerebral magnetic resonance imaging and other further CSF tests could not be done at this hospital. After 18 days of routine antiviral treatment (0.1 g ibavirin per day for 7 days), immunoglobulin therapy (10 g per day for 5 days), and antipsychotic drug treatment, the neurological and mental abnormalities were greatly ameliorated. He was discharged with mild irritability, slight shaking of the hands, and walking fatigue. These symptoms have persisted up until our last follow-up (May 4, 2020).

## 3 Discussion

It is speculated that coronaviruses (SARS and MERS) may affect the central nervous system in 1 of 2 ways: 1 is by spreading from the peripheral to the central nervous system through nerve fibers, and the other is by passing through the blood-brain barrier.^[1–3]^ Neurological complications do not appear concomitantly with respiratory symptoms; they are usually delayed 2 to 3 weeks in patients infected with coronaviruses. Neurological symptoms in patients with COVID-19 are grouped into several categories, including those related to acute cerebrovascular disease, intracranial infection, peripheral nervous system symptoms, and neuromuscular symptoms.^[2,7–10]^ Nevertheless, it was reported that some patients with neurological symptoms suspected to have COVID-19 could not be diagnosed clearly because of negative RNA test results using blood and CSF samples.^[11]^ The clinical data on COVID-19 infection and neurological damage are still very limited.

We believe this is the first case of central and peripheral neurological damage and mental abnormalities with confirmed COVID-19 infection by CSF antibody tests. This patient showed aggressive neurologic damage and mental abnormalities without severe respiratory symptoms. There was no obvious point in time when neurological damage began, and the neurological damage lasted for months. Craniocerebral CT images were normal, and SARS-CoV-2 antibody titers were 100 times higher in the CSF than in serum with negative RNA test results.

The negative RNA tests in this case indicate a low level of virus replication, and strong positive antibodies and high protein level in the CSF are suggestive of autoimmune encephalitis or encephalopathy secondary to coronavirus infection. The neurologic symptoms of this patient were possibly induced by the immune response to infection rather than due to direct virus damage. However, more answers need to be uncovered, including

(1) understanding who is susceptible to this kind of neurological damage,
(2) whether psychiatric abnormalities are common in patients with COVID-19 encephalitis,
(3) whether this patient's fatigue was directly caused by encephalitis or combined with an impaired peripheral nervous system,
(4) whether antibody tests are an essential exam for CSF tests as well as RNA tests,
(5) whether regular immunoglobulin therapy for COVID-19 could prevent and delay the development of neurologic damage in general, and
(6) the possible short- and long-term harm to the nervous system by COVID-19.

Further studies must be conducted to investigate the neurological damage caused by this novel coronavirus.

We suggest that physicians who encounter patients from pandemic hotspots with abnormal neurologic damage and mental abnormalities protect themselves carefully, even if the RNA test for the virus shows negative results. Antibody tests for CSF as well as RNA tests are necessary for patients with neurological symptoms, and long-term follow-up is recommended for these patients. The findings of this case are important for the assessment and follow-up of COVID-19 patients.

## Author contributions

W.M. and F.Q responded for the care of the patient, W.M. W.L. and Y.G. were involved in the diagnosis, management and treatment. Y.G. and T.L. reviewed relevant papers. All authors contributed equally to writing the manuscript.
